# Adaptation of families of adult patients with brain tumor: Partial least squares structural equation modeling

**DOI:** 10.1371/journal.pone.0285677

**Published:** 2023-05-11

**Authors:** Mijung Jung, Younhee Jeong, Bong Jin Park

**Affiliations:** 1 Department of Nursing, Kwangju Women’s University, Gwangju, Korea; 2 College of Nursing Science, Kyung Hee University, Seoul, Korea; 3 East-West Nursing Research Institute, Kyung Hee University, Seoul, Korea; 4 College of Medicine, Kyung Hee University, Seoul, Korea; 5 Department of Neurosurgery, Kyung Hee Medical Center, Seoul, Korea; University of Almeria: Universidad de Almeria, SPAIN

## Abstract

Brain tumor patients experience physical, psychological, social, and cognitive changes. These changes are challenging for both the patients and their families. These patients and their families need to adapt together on the cancer treatment path. This study aimed to identify the factors affecting adaptation in families of adult patients with brain tumors. A quantitative, cross-sectional study of 165 families of adult patients with primary brain tumors was conducted using a self-administered questionnaire. Partial least squares structural equation modeling was used to test a hypothetical model. The results showed that family stress, family functioning, and family resources influenced on family adaptation in families of adult patients with primary brain tumors. Among these factors, family resources were identified to be the strongest factor associated with family adaptation. The results of this study may be utilized as a theoretical basis in nursing to improve the family adaptation of patients with brain tumors. Regarding nursing practices, the results suggest that nurses should provide family-centered nursing interventions and promote family resources to help brain tumor patients and their families to adapt.

## Introduction

Cancer is one of the most feared chronic diseases, and brain tumors are the most frightening of all forms of cancer because brain tumors are difficult to treat and cause comprehensive systemic effects due to neurological damage, compared to other cancers [1, 2]. In addition to the impact of the tumor itself, patients with brain tumors may experience physical, psychological, social, and cognitive changes during and after treatment as well [3–6]. These changes are challenging for patients to overcome alone, and their families are also burdened and stressed [7]. The family’s burden and stress ultimately affect the patient’s health and their likelihood of survival [8]. Therefore, it is more effective to approach the family as a unit, and family adaptation is as important an outcome as patient care.

To promote family adaptation, understanding factors affecting family adaptation is crucial in the families of patients with brain tumors. One of the commonly applied theories on family adaptation is the resiliency model of family stress, adjustment, and adaptation [9], which considers family resilience as a key concept to enhance family adaptation. Family functioning, situational appraisal, family resources, and problem-solving and coping are factors mediating resilience during the adaptation phase in the model. This model has been used in previous studies on families with adult patients with dementia, post-traumatic stress disorder, and congestive heart failure [10–12]. Although these studies declared that their studies were based on the resiliency model, either the focus of the studies was family burden rather than family adaptation, or the various associations between factors were not examined. In addition, the resiliency model was not tested in families of adult patients with brain tumor.

**Fig 1:**
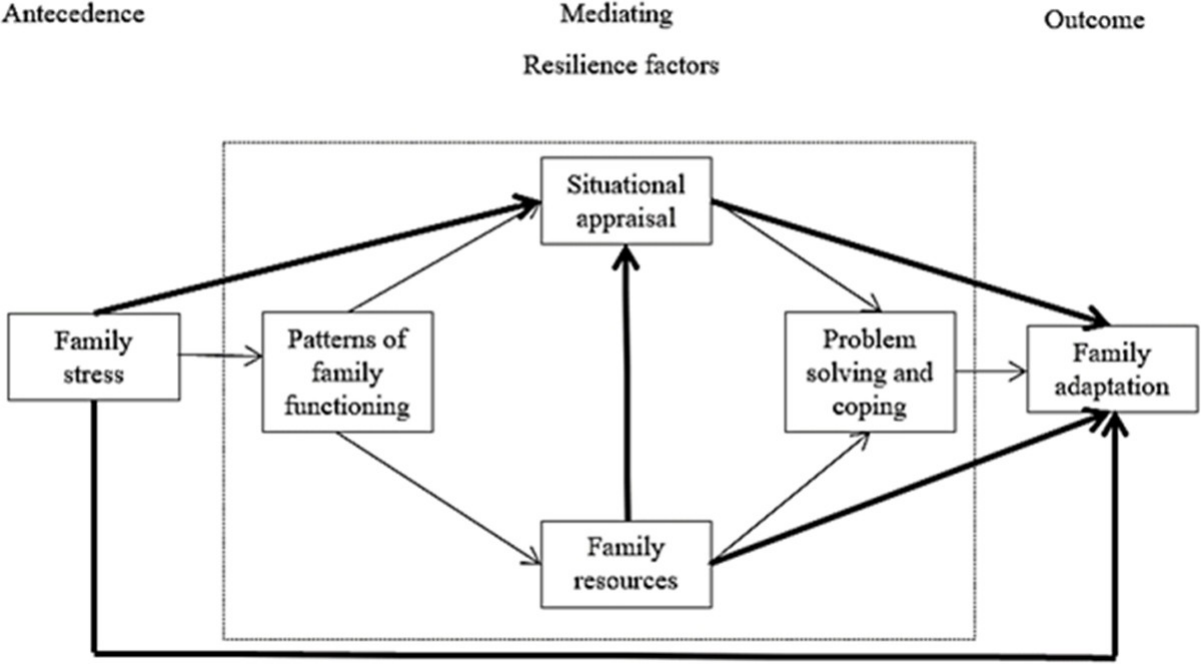
Hypothetical model. The thick lines in this model are the added paths to the Family Resilience Model.

There have been studies on brain tumor patients regarding family stress, negative emotions, and family management patterns [13, 14]. However, those studies included only psychological distress or family functioning. It is necessary to expand studies on families of brain tumor patients to include more comprehensive resilience factors influencing family adaptation. Resilience as a characteristics, dimensions, and property of families helps such families be resistant to disruption in the face of change and adaptive in the face of crisis situation [15]. Therefore, this study explored the factors influencing adaptation in families of adult brain tumor patients based on the concepts of the resiliency model of family stress, adjustment, and adaptation. Based on the model of McCubbin et al. [9], we have built a hypothetical model consisting of a total of 11 paths by adding five paths through literature reviews [10, 16–20] and tested the hypothetical model (Fig 1). Mellon et al. [19] found that more concurrent family stressors reported more negative appraisal of the cancer, and that family resources also affected appraisal of the cancer. Kim et al. [18] reported that family stress had a direct effect on family adaptation in the family members of elderly patients with dementia. In addition, family resources had a positive correlation with situational appraisal [21], and situational appraisal and family resources were positively related to family adaptation [22].

We used partial least squares structural equation modeling (PLS-SEM) to validate our model. PLS-SEM is useful to predict main constructs and to extend existing theory, and it does not necessarily require normality assumption of the data [23]. In addition, PLS-SEM is useful for exploratory research [24].

This study aimed to extend the resiliency model of family stress, adjustment, and adaptation as well as predict the adaptation of families of primary brain tumor patients. This is the first study to apply a modified resiliency model to families of brain tumor patients. The study should contribute to clinical family care for brain tumor patients by modifying the family care model scientifically. The result of this study also highlights that nurses should provide integrated nursing to help families with brain tumor patients for more positive family adaptation.

## Methods

### Design and participants

The design of this study was a cross-sectional survey. The participants of this study were the families of adult patients with primary brain tumors. First, we identified patients who met the selection criteria through a medical records review, and family members who accompanied these patients when receiving outpatient treatment were the participants in the study. The criteria for participants (family members) were (a) being an adult 18 years old or over and (b) understanding the research and agreeing to participate in this research. The criteria for patients were (a) being an adult 18 years old or over, (b) being diagnosed with a primary brain tumor, (c) the tumor not having metastasized to other organs, and (d) having completed the acute phase of treatment and having visited the outpatient clinic for further management and follow-up.

An a priori sample size calculation was conducted using the a-priori sample size calculator for structural equation models by Soper [25]. The sample size of 161 was calculated based on a power level of 0.8, a probability level of 0.05, an effect size of 0.3, six latent variables, and 12 observed variables. Considering the missing rate (10%), 178 participants were recruited from two university hospitals in Seoul, Korea. Thirteen responses were excluded from the final analysis due to incomplete or missing data; therefore, the data for 165 participants were included in the final analysis.

### Ethical approval

The institutional review boards of two university hospitals (No. 2018-01-060-002 and No. 2018-01-018-003) approved the study. Informed written consent was obtained from all family members and patients who participated in this study after being informed of the purpose of the study and given a full explanation of the process. We also explained that the participants were allowed to withdraw from the study at any time.

### Measurements

The participants’ general characteristics and the variables included in the hypothetical model were measured using self-report questionnaires. The disease-related characteristics were assessed through electronic medical records at the two hospitals. All tools were approved for use and modification by the developers. The questionnaires that were not previously translated into Korean were translated through the translation and back translation process by a professional translator and a bilingual nursing researcher. Prior to conducting the survey, the translated questionnaires were tested on lay-persons to confirm if the meanings were understandable. The translated version of the questionnaires was finalized after the sentences that were difficult to understand for lay persons were changed to easily understandable expression.

Upon development, all measurement tools were incorporated into the reflective model. We used this reflective model to measure each latent variable. The reflective model is a type of measurement model setup in which the direction of the arrow is from the construct to the indicator variables, indicating the assumption that the construct causes the measurement of the indicator variables [26].

#### Family stress

Family stress was measured by the family stressors index and the family strains index [9]. The questionnaires consisted of questions with “yes/no” responses. “Yes” responses were converted to weighted scores, and the total scores were divided by the number of items identified for final family stressor scores and family strain scores as recommended by McCubbin and Patterson [9]. The higher the score, the greater the family stress.

#### Family functioning

The Family Attachment and Changeability Index 8 (FACI8) developed by McCubbin, et al. [27]. Thompson was used to measure family functioning. The FACI8 consists of 16 items and a five-point Likert-type scale with two domains (attachment and changeability). Higher scores reflect higher family function. The alpha reliability of the scale in this study was .84.

#### Situation appraisal

The Family Hardiness Index (FHI), developed by McCubbin, et al. [28] and translated by Sim [29], was used to measure situational appraisal. The tool consists of 20 items with a four-point Likert-type scale and three domains: commitment, challenge, and control. The higher the score, the more positively the family appraises the situation. The alpha reliability of the scale in this study was .86.

#### Family resource

The Family Inventory of Resources for Management (FIRM), developed by McCubbin, Comeau, & Harkins [15] and translated and modified by Kim [18], was used to assess family resources. The modified FIRM consisted of 20 items with a four-point Likert-type scale and four domains (esteem and communication, mastery and health, financial well-being, and social desirability). The higher the score, the greater the family resources. The alpha reliability of the scale in this study was 87.

#### Problem-solving and coping

The Family Crisis Oriented Personal Evaluation Scales (F-COPES), developed by McCubbin, Larsen, & Olson [30] and translated and modified by Seomun [31], was used to assess family problem-solving and coping. The scales are composed of 20 items with a five-point Likert scale and two domains (reframing and seeking spiritual support). The higher the scores, the greater the resources the family has. The alpha reliability of the scale in this study was .79.

#### Family adaptation

The Family Member Well-Being Index (FMWBI), developed by McCubbin and Patterson [32] and translated and modified by Seomun [31], was used to assess family adaptation. The index is comprised of eight items with a 10-point Likert scale. A higher score indicates better family adaptation. The alpha reliability of the index in this study was .90.

### Data collection

A researcher and research assistants collected data at the neurosurgical outpatient clinics at the two university medical centers from February 19 to August 29, 2018. The research assistants were nurses trained for the data collection process and the use of the questionnaires prior to the survey for interrater reliability. The researcher and research assistants explained thepurpose of the study and provided information to the family members and patients. Upon completion of the questionnaires, disease-related information about the patients and the absence of metastasis were checked through electronic medical records of the two hospitals.

### Data analysis

IBM SPSS Statistics, version 25.0 (IBM, Chicago, IL, USA) and SmartPLS 3.3.3 (SmartPLS GmbH, Boenningstedt, Germany) were used for analysis. Data were summarized using means and standard deviations. Correlations between measured variables were analyzed using the Pearson correlation coefficient. PLS-SEM was used to test the hypothetical model. To validate the measurement model, internal consistency reliability, convergent validity, and discriminant validity were tested by composite reliability (CR), average variance extracted (AVE), and the criterion of Fornell-Larcker [33], respectively. The methods of structural model validation are different in PLS-SEM [34]. The coefficient of determination *R*^2^, predictive relevance *Q*^2^, and effective size of path coefficients (*f*^2^) were used to validate the model. The conditions for using the PLS algorithm and bootstrapping were set as follows: maximum number of iterations = 1,000, stopping criterion = 1 × 10^−7^, a sample size of 5,000 for bootstrapping, and a significance of 0.05.

## Results

### Participant and patient characteristics

Participant characteristics are shown in Tables 1 and 2. Family caregivers were 84 men (50.9%) and 81 women (49.1%), with an average age of 53 years, while the patients consisted of 51 men (30.9%) and 114 women (69.1%), with an average age of 64 years. Most patients had meningioma (52.7%) or pituitary adenoma (20.0%) as the primary brain tumor. Most of the participants were living with the patient.

**Table 1:** Demographic characteristics of the family caregiver. (*N* = 165).

Variable	Categories	n	(%)	Mean ± SD
**Age (years)**	20–29	11	(6.7)	53.33±14.56
	30–39	19	(11.5)	
	40–49	31	(18.8)	
	50–59	44	(26.7)	
	≥60	60	(36.4)	
**Gender**	Female	81	(49.1)	
	Male	84	(50.9)	
**Relationship to the patient**	Parents	11	(6.7)	
	Sibling	9	(5.5)	
	Spouse	76	(46.1)	
	Offspring	57	(34.5)	
	Daughters-in-law	6	(3.6)	
	Others	6	(3.6)	
**Cohabitation**	Yes	113	(68.5)	
	No	51	(30.9)	
	No response	1	(0.6)	
**Level of education**	Elementary school	15	(9.1)	
	Middle school	19	(11.5)	
	High school	55	(33.3)	
	College or above	70	(42.4)	
	Others	6	(3.6)	
**Monthly family income (1,000 won)**	<2,000	39	(23.6)	
	2,000–3,999	61	(37.0)	
	≥4,000	58	(35.2)	
	No answer	7	(4.2)	
**Religion**	Christian	40	(24.2)	
	Catholic	22	(13.3)	
	Buddhist	27	(16.4)	
	Others	3	(1.8)	
	None	73	(44.2)	

Abbreviations: SD, standard deviation; OP, operation; RT, radiation therapy; CT, chemotherapy

**Table 2:** Demographic and disease-related characteristics of the patients. (*N* = 165).

Variable	Categories	n	(%)	Mean ±SD
**Age (years)**	20–29	6	(3.6)	63.99 ±13.90
	30–39	6	(3.6)	
		
	40–49	11	(6.7)	
	50–59	22	(13.3)	
	≥60	120	(72.7)	
**Gender**	Female	114	(69.1)	
	Male	51	(30.9)	
**Diagnoses**	Meningioma	87	(52.7)	
	Lymphoma	2	(1.2)	
	Pituitary adenoma	33	(20.0)	
	Schwannoma	19	(11.5)	
	Hemangioma	2	(1.2)	
	Germinoma	3	(1.8)	
	Craniopharyngioma	2	(1.2)	
	Glioblastoma	9	(5.5)	
	Glioma	2	(1.2)	
	Oligodendroglioma	3	(1.8)	
	Astrocytoma	3	(1.8)	
**Duration of the disease (months)**	<12	32	(19.4)	66.78 ±73.80
	12–23	29	(17.6)	
	24–59	41	(24.8)	
	≥60	63	(38.2)	
**Treatment Modality**	Conservative -management	7	(4.2)	
	OP	137	(83.0)	
	RT	3	(1.8)	
	OP and RT	11	(6.7)	
	OP, CT, and RT	7	(4.2)	

Abbreviations: SD, standard deviation; OP, operation; RT, radiation therapy; CT, chemotherapy

### Data normality and multicollinearity

The descriptive statistics of the measured variables are shown in Table 3. The absolute values of kurtosis and skewness were below 2 and 7, respectively. Thus, the normality of each variable was appropriate However, the multivariate normality assumption was not met according to Mardia’s multivariate coefficient (17.27, *p* < .05). The correlations between variables are also shown in Table 3. The absolute values of correlation coefficients were not greater than .80, and the variance inflation factor (VIF) values were less than 5. Therefore, there was no multicollinearity issue.

**Table 3:** Descriptive statistics and correlation (*r*) for measured variables.

	Y12	X1	X2	Y1	Y2	Y3	Y4	Y5	Y6	Y7	Y8	Y9	Y10	Y11	Mean	SD	Skewness	Kurtosis
**Y12**	1														6.02	2.10	-0.46	-0.18
**X1**	-.16^*^	1													10.65	8.85	0.83	0.37
**X2**	-.32^**^	.40^**^	1												17.32	13.96	0.36	-1.24
**Y1**	.33^**^	-.28^**^	-.53^**^	1											4.00	0.68	-0.42	-0.45
**Y2**	.38^**^	.02	-.34^**^	.50^**^	1										3.63	0.75	-0.02	-0.19
**Y3**	.32^**^	-.04	-.35^**^	.47^**^	.65^**^	1									3.27	0.47	-0.33	2.00
**Y4**	.25^**^	.11	-.05	.23^**^	.49^**^	.55^**^	1								2.88	0.53	-0.44	1.32
**Y5**	.36^**^	-.18^*^	-.37^**^	.55^**^	.36^**^	.43^**^	.20^*^	1							3.34	0.56	-0.77	0.50
**Y6**	.44^**^	.01	-.22^**^	.41^**^	.65^**^	.76^**^	.69^**^	.36^**^	1						3.94	0.63	-0.99	3.18
**Y7**	.37^**^	-.08	-.31^**^	.47^**^	.55^**^	.70^**^	.51^**^	.28^**^	.68^**^	1					3.86	0.72	-0.60	1.01
**Y8**	.51^**^	-.16^*^	-.31^**^	.31^**^	.33^**^	.35^**^	.23^**^	.36^**^	.41^**^	.30^**^	1				3.31	1.02	-0.57	-0.21
**Y9**	.30^**^	-.18^*^	-.35^**^	.55^**^	.52^**^	.56^**^	.45^**^	.33^**^	.61^**^	.60^**^	.27^**^	1			3.48	0.76	-0.17	0.00
**Y10**	.36^**^	-.11	-.23^**^	.32^**^	.52^**^	.61^**^	.46^**^	.40^**^	.69^**^	.52^**^	.40^**^	.42^**^	1		3.75	0.61	-0.11	0.51
**Y11**	.09	-.04	-.02	.01	.01	.08	.19^*^	.10	.11	.15	-.01	.19^*^	.16^*^	1	2.34	1.25	0.56	-0.90

Abbreviations: X1, Family stressor; X2, Family strain; Y1, Attachment; Y2, Changeability; Y3, Commitment; Y4, Challenge; Y5, Control; Y6, Esteem and communication; Y7, Mastery and health; Y8, Financial well-being; Y9, Social desirability; Y10, Reframing; Y11, Seeking spiritual support; Y12, Family adaptation.

**p < .01, *p <. 05

**p < .01, *p <. 05

### Measurement model

We validated the measurement model (the outer model). The results of the measurement model validation are presented in Table 4. According to Hair et al. [34], composite reliability (CR) is a better measure of internal consistency. The CRs were between .67 and .86. All the values were greater than .60; therefore, internal consistency reliability was acceptable [35]. The AVEs of constructs ranged from .54 to .75, which were over .50. Thus, convergent validity was confirmed. Because the squared roots of the AVEs were approximately equivalent or higher than the inter-construct correlations, discriminant validity was confirmed as well. Accordingly, the measurement model met all the criteria for hypothesis testing.

**Table 4:** Convergent and discriminant validity evaluation of measurement model.

	FA	FR	FS	PFF	PSC	SA	CR	AVE
**FA**	***1***						1	1
**FR**	.50	**.*79***					0.86	0.62
**FS**	-.32	-.35	**.*81***				0.78	0.66
**PFF**	.41	.71	-.47	**.*86***			0.86	0.75
**PSC**	.36	.66	-.22	.46	**.*74***		0.67	0.54
**SA**	.39	.80	-.30	.69	.64	**.*77***	0.82	0.60

Abbreviations: FA, family adaptation; FR, family resources; FS, family stress; PFF, patterns of family functioning; PSC, problem-solving and coping; SA, situational appraisal

**Note.** The squared root of AVE is presented in bold and italic.

### Structural model

#### Predictive accuracy

**Fig 2:**
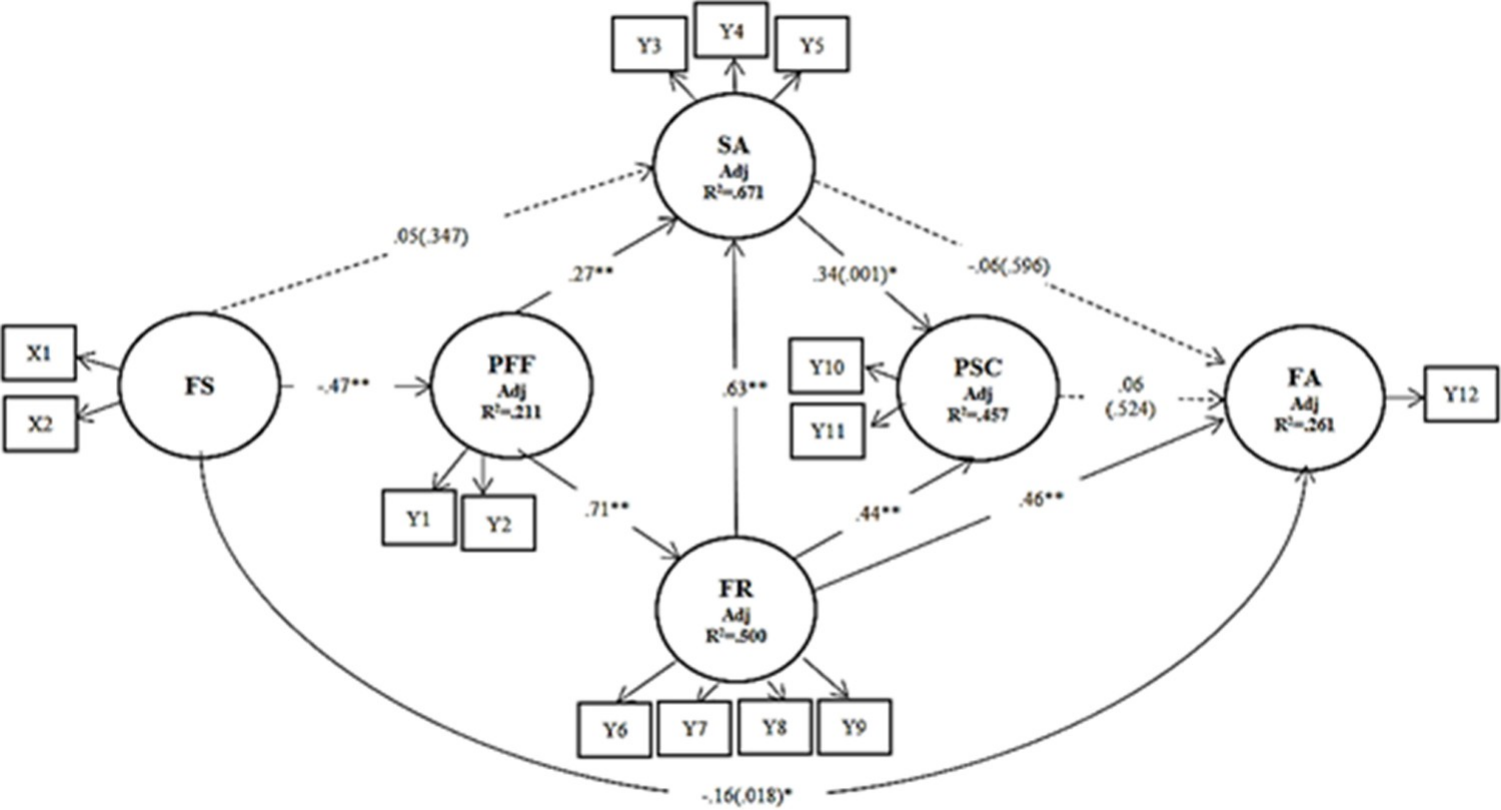
Path diagram of the model. X1, Family stressor; X2, Family strain; Y1, Attachment; Y2, Changeability; Y3, Commitment; Y4, Challenge; Y5, Control; Y6, Esteem and communication; Y7, Mastery and health; Y8, Financial well-being; Y9, Social desirability; Y10, Reframing; Y11, Seeking spiritual support; Y12, Family adaptation. **p < .01; * *p*< .05.

To validate our structural model (inner model), we evaluated coefficients of determination *R*^2^ for the amount of variance accounted for in the construct, also called prediction accuracy. The structural model validation is presented in Table 5. Eight out of 11 paths were statistically significant, while three paths were not (Fig 2). The final model explained 26.1% of the variance in family adaptation, 67.0% of the variance in situational appraisal, 45.7% of the variance of problem-solving and coping, 50.0% of the variance in family resources, and 21.1% of the variance in family functioning (Fig 2 and Table 5).

**Table 5:** Model fit for the hypothetical model.

Latent variables	*R* ^*2*^	Adj *R*^*2*^	*Q²*
**Patterns of family functioning**	.215	.211	0.16
**Situation appraisal**	.677	.671	0.39
**Family resources**	.503	.500	0.30
**Problem-solving and coping**	.467	.457	0.24
**Family adaptation**	.279	.261	0.21

#### Predictive relevance

*Q*^2^ is a mechanism for assessing the structural model and its predictive relevance [36]. The cross-validated redundancy measure values (*Q*^2^) of all constructs were larger than zero (Table 5). *Q*^2^ greater than zero indicates that the model has predictive fitness for a particular construct [26]. Therefore, our model presented predictive relevance.

#### Effect size

PLS-SEM estimates the importance of the exogenous constructs in explaining the endogenous construct, and it re-calculates *R*^2^ by omitting one exogenous construct at a time [37]. In our model, the paths with large effect size were paths between family functioning and family resources (*f*^2^ = 1.011) and between family resources and situational appraisal (*f*^2^ = .612). The path between family stress and family functioning showed a medium effect size (*f*^2^ = .274). The rest of the relationships showed a small effect size (Table 6). The effect size of path coefficients (multiple partial correlation, *f*^2^) higher than .35 were classified as large effect size, and those between .15 and .35 were classified as medium effect size, following [38].

**Table 6:** Standardized direct, indirect, and total effects for the model.

Endogenous variables	Exogenous variables	*f* ^*2*^	Standardized direct effects (*p*)	CI 2.5%	CI 97.5%	Standardized indirect effects (*p*)	CI 2.5%	CI 97.5%	Standardized total effects (*p*)	CI 2.5%	CI 97.5%
**Patterns of family functioning**	Family stress	.274	-.47 (< .001)	-.568	-.356				-.46 (< .001)	-.568	-.356
**Situational appraisal**	Family stress	.005	.05 (.347)	-.055	.137	-.33 (< .001)	-.431	-.239	-.28 (< .001)	-.411	-.163
	Patterns of family functioning	.097	.27 (< .001)	.120	.396	.45 (< .001)	.345	.564	.71 (< .001)	.629	.794
	Family resources	.612	.63 (< .001)	.502	.754				.63 (< .001)	.502	.754
**Family resources**	Family stress					-.33 (< .001)	-.424	-.242	-.33 (< .001)	-.424	-.242
	Patterns of family functioning	1.011	.71 (< .001)	.636	.778				.71 (< .001)	.636	.778
**Problem-solving and coping**	Family stress					-.20 (< .001)	-.298	-.122	-.20 (< .001)	-.298	-.122
	Patterns of family functioning	.007				.56 (< .001)	.427	.697	.47 (< .001)	.347	.591
	Situational appraisal	.071	.34 (.001)	.124	.533				.34 (.001)	.124	.533
	Family resources	.115	.44 (< .001)	.247	.655	.22 (.001)	.080	.339	.66 (< .001)	.497	.808
**Family adaptation**	Family stress	.033	-.16 (.018)	-.299	-.027	-.15 (< .001)	-.219	-.082	-.31 (< .001)	-.428	-.186
	Patterns of family functioning					.31 (< .001)	.186	.421	.31 (< .001)	.186	.421
	Situational appraisal	.002	-.06 (.596)	-.273	.170	.02 (.545)	-.042	.090	-.04 (.713)	-.247	.187
	Family resources	.089	.46 (< .001)	.193	.689	.00 (.999)	-.163	.179	.46 (< .001)	.290	.599
	Problem-solving and coping	.003	.06 (.524)	.119	.242				.06 (.525)	-.119	.242

Direct effect is the direct relationship between an exogenous and an endogenous latent variable while the indirect effect represents a relationship between an exogenous and an endogenous latent variable via a third construct in the PLS path model [39]. Eight out of 11 paths in the structural model were statistically significant at *p* < .05, while three paths were not. The standardized path coefficients are presented in Fig 2 and Table 6. Family stress and family resources directly influenced family adaptation (*ß* = -.16, *p* = .018; *ß* = .46, *p* < .001, respectively), while family functioning had an indirect impact on family adaptation through family resources (*ß* = .31, *p* < .001). Situational appraisal (*ß* = -.04, *p* = .713), as well as problem-solving and coping (*ß* = .06, *p* = .525), were not associated with family adaptation either directly or indirectly.

**Fig 3:**
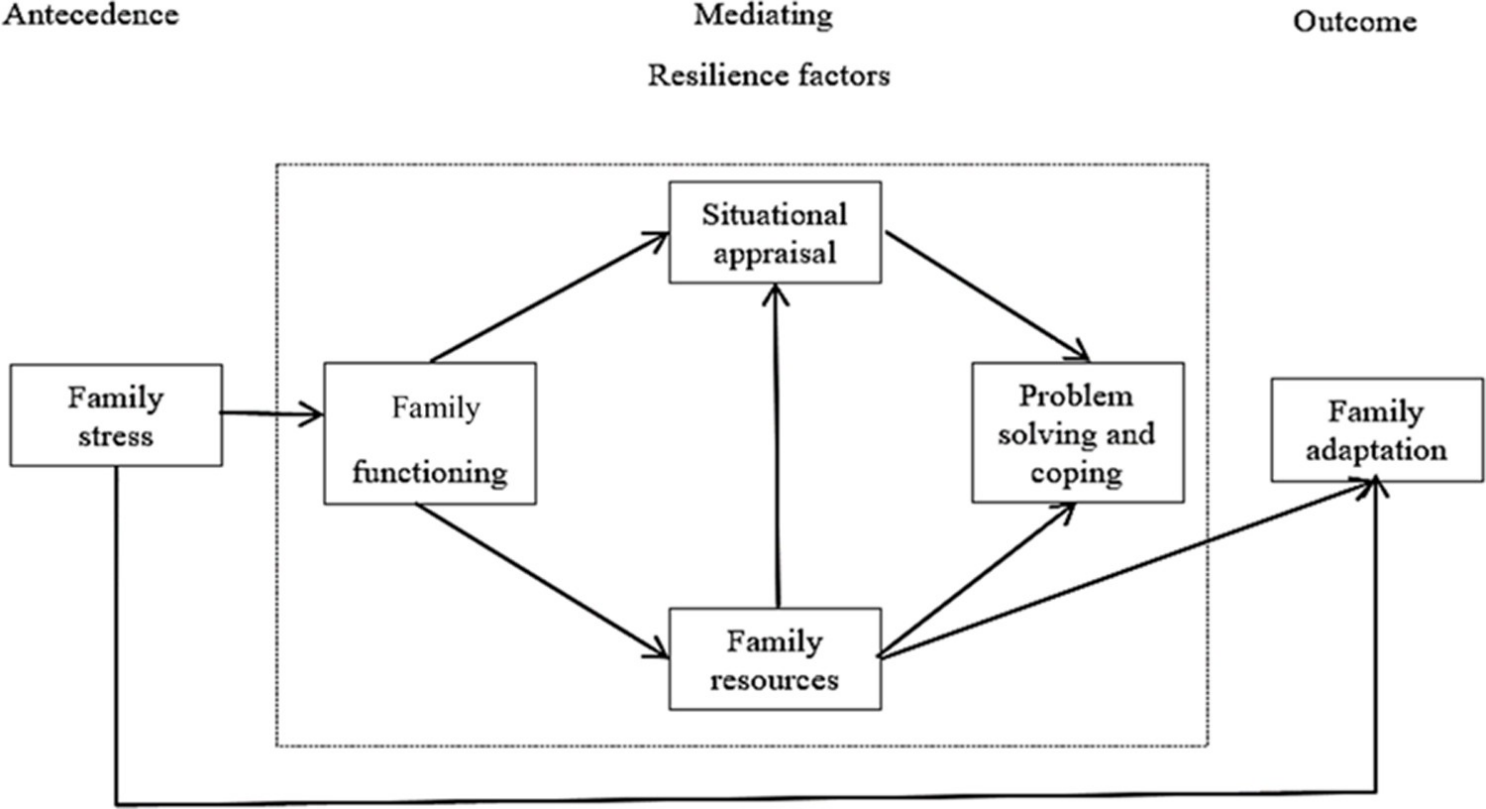
The significant paths of the model.

Family resources had an impact on situational appraisal (*ß* = .63, *p* < .001) and problem-solving and coping (*ß* = .66, *p* < .001). Family functioning had direct associations with situational appraisal (*ß* = .27, *p* < .001) and family resources (*ß* = .71, *p* < .001), while family stress had an indirect association with situational appraisal (*ß* = -.33, *p* < .001), family resources. *(ß* = -.20, *p* < .001), and family adaptation (*ß* = -.15, *p* < .001). A simplified model including the eight significant paths only is shown in Fig 3.

## Discussion

This study was performed to identify the factors affecting adaptation in families of adult patients with brain tumors using a hypothetical model based on the resiliency model of family adjustment and adaptation [9]. In this model, the main predictors explained 26.1% of the variance in family adaptation in families of adult patients with brain tumors. The results showed that family stress, family functioning, and family resources were significant factors affecting family adaptation of families of brain tumor patients. Among the predictors, family resources had the greatest effect on family adaptation and played an important mediating role between family stress and adaptation.

Brain tumor patients and their families experience multidimensional stress [40]. Depending on location of the brain tumor, limitations in functional activities of daily living (ADL) such as bathing, dressing, toileting, transferring, and feeding occur and cause dependence of patients on their caregivers. The higher the patient’s dependence on the family caregiver to perform ADL, the higher the burden on the family [40]. In addition, the neuropsychiatric symptoms of the patients had a significant impact on caregiver burden [41]. The patients and family caregivers suffered from psychosocial distress and financial burden [40, 42, 43]. Thus, a problem for one family member becomes a problem for the whole family. Family stress, especially caregiver burden, also makes an impact on lowering the cancer patient survival rate [8]. Therefore, it is important for nurses to reduce cancer patients’ family stress by early detection and intervention, and to help them to adapt. To reduce family stress and improve family adaptation, family resilience is important [44], given that resilience is a protective factor for family [45]. Our study showed that family stress had a negative effect on family adaptation, which is consistent with previous studies on the families of patients with chronic illnesses [18, 46, 47]. Thus, when nurses provide nursing care to patients with brain tumors in the clinical field, they should consider what kinds of stress the families of patient experience and find various interventions to relieve their stress.

In addition, the results showed that the impact of family stress on family adaptation was mediated by family functioning and family resources. Family functioning and family resources are major resilience factors of adaptation in the resiliency model of family adjustment and adaptation [9]. In this study, family functioning was directly affected by family stress, suggesting that family stress decreases family functioning. This is consistent with earlier results that showed that there was an association between family functioning and family stress [48, 49]. Additionally there was an association between family functioning and family adaptation [50]. In our study, family functioning had no direct effect on family adaptation but mediated the role of family stress on family resources, which had a direct relationship with family adaption. Therefore, an intervention to improve family functioning is needed to help families of patients with brain tumors to eventually adapt well.

Family functioning is a multidimensional concept, and multidisciplinary approaches are needed to improve it [51]. According to the study of Lee and Yun [52], family participation in decision-making and communication are associated with family functioning. In addition, more adaptive family functioning (cohesiveness, effective communication, low conflict) is associated with better cancer patient’s outcomes and better quality of life [53]. Moreover, the family can resolve the problem more effectively through good family functioning [54]. Therefore, it is recommended for nurses to support families in communicating effectively and participating in the cancer treatment process as a unit for better family functioning.

The present results imply that family resources are critical for family adaptation. This is consistent with previous studies showing that family resources lessen the burden on the family and facilitate family adaptation [55, 56]. In this study, family resources consist of four subcategories: esteem and communication, mastery and health, financial well-being, and social desirability. For families of patients with brain tumors, these four sub-factors should be met as resources. Therefore, health care professionals should consider family esteem, communication, mastery, and social desirability for improving family resources. Also, nurses should identify the financial situation of the family of brain tumor patients and, if necessary, support them to receive financial support from the government or community because lower socioeconomic status has been reported to influence caregiver burden [41].

Various family resources have previously been examined in relation to family stress and family adaptation, but social support has mainly been studied [7, 57]. For successful management of support groups for brain cancer patients and their families, it is necessary to assess benefits and barriers [58]. Nurses should have the initiative to provide support group interventions and be involved in developing and maintaining brain tumor support groups [59]. An interesting nurse-led support group intervention, the so-called FamilyStrong program, was reported recently [60]. The FamilyStrong program is a clinic-based telehealth support service, but the effectiveness of the intervention has not yet been reported. The program appears to be useful as a support system, especially in this pandemic era. Psychosocial care and information are also reported to be effective ways of improving family resources [61, 62]. In addition, the government need to actively consider providing a respite care program to alleviate the burden on the families of brain tumor patients [63].

In this study, situational appraisal as well as problem-solving and coping had no direct effect on family adaptation. First, the relationship between problem-solving and coping and family adaptation was different compared to the relationship in the McCubbin’s resiliency model [9]. The subcategories of problem-solving and coping were reframing and seeking spiritual support. Since most of the participants did not have a religion, it is assumed that the questions of problem-solving and coping were interpreted in terms of religion. Therefore, it is necessary to conduct repetitive research by expanding the areas with more participants. Second, the situational appraisal as well as problem solving and coping did not have a significant direct, indirect, or total effect on family adaptation. Thus, the results of this study support the original model, which suggested that situational appraisal and family adaptation have no direct effect.

## Limitations and implications

There are some limitations to consider in this study. The survey was done by convenience sampling at two university medical centers. Since the primary brain tumor patients were selected through only two hospitals among all patients in the country, further studies are needed with a larger sample size. This study was also conducted with a cross-sectional design. Longitudinal studies are needed in the future to identify progress over time of family adaptation in families of patients with brain tumors.

Despite its limitations, this study has several clinical nursing implications. First, this study examined factors affecting family adaptation of the families of adult brain tumor patients based on McCubbin’s resiliency model of family adjustment and adaptation. To our knowledge, this is the first study to apply McCubbin’s resiliency model to the families of adult patients with primary brain tumors. In addition, we examined the possibility of extension of the model by testing additional paths. Second, the results of this study may be used as a theoretical basis for evidence-based nursing to improve family adaptation in families of patients with brain tumors in clinical settings. Third, we utilized PLS-SEM for analyzing the data, which is an effective predictive analysis, to examine the effect of exogenous variables on endogenous variables. Third, we utilized PLS-SEM for analyzing the data, which is an effective predictive analysis, to examine the effect of exogenous variables on endogenous variables. Fourth, based on the results of this study, more integrated nursing care can be provided if the family is included in the nursing of brain tumor patients and nursing care is provided considering family stress, family functioning, and family resources.

## Supporting information

S1 Dataset (XLSX)
